# 2 km Uncompressed HD Video Wireless Transmission at 100 GHz Based on All-Optical Frequency Up- and Down-Conversion

**DOI:** 10.3390/mi15121488

**Published:** 2024-12-11

**Authors:** Shuang Gao, Yutong Jiang, Zhuoxin Li, Qing Zhong, Min Zhu, Jiao Zhang

**Affiliations:** 1 National Mobile Communications Research Laboratory, Southeast University, Nanjing 210096, China; shuang_gao@seu.edu.cn (S.G.); qingzhong@seu.edu.cn (Q.Z.); 2 Purple Mountain Laboratories, Nanjing 211111, China

**Keywords:** millimeter-wave, all-optical transceiver, uncompressed HD video transmission

## Abstract

The millimeter-wave wireless transmission system is widely regarded as a promising solution for applications of future 6G communication. This paper presents an experimental comparison between all-optical and all-electric receivers for millimeter-wave communication systems over a 15 m wireless link and demonstrates 200 m and 2 km real-time uncompressed HD video transmission using an all-optical transceiver at 100 GHz. The systems leverage photonics-assisted heterodyne beating techniques at the transmitter, while the receivers employ either an avalanche photodiode (APD)-based all-optical approach or an envelope detection-based all-electric approach. Experimental results show that the all-optical transceiver supports significantly higher transmission rates, achieving error-free transmission at up to 11.318 Gbps over a 200 m wireless link without clock recovery, compared to the all-electric receiver, which is limited to only 3.125 Gbps error-free 15 m transmission. This work proves that the proposed system based on the all-optical receiver is more promising for supporting future 6G scenarios requiring ultra-wideband, high capacity, and wide coverage high-speed wireless communications.

## 1. Introduction

The millimeter-wave (mmWave) band has garnered significant attention for its high bandwidth efficiency, low latency, and ability to meet the demands of emerging bandwidth-hungry applications [1,2]. At the transmitting side of mmWave wireless communication systems, to convert the baseband data signal into the mmWave frequency band, there are typically two technical approaches to achieve mmWave up-conversion. One is the all-solid-state electronic mixing technique, and the other is the photonics-assisted heterodyne beating technique. The photonics-assisted technique leverages the advantages of optical devices, such as their wide bandwidth and high-frequency response, which has demonstrated potential in overcoming the limitations of conventional electronic devices, enabling ultra-wideband communication with higher data rates and better transmission performance [3]. In addition, mmWave up-conversion based on the photonics-assisted technique has been widely used due to its convenience in facilitating fiber-wireless-fiber (FiWiFi) convergence. At the receiving side of mmWave wireless communication systems, all-electric receivers based on envelope detection and all-optical receivers are two main categories for mmWave down-conversion approaches. Unlike all-electric receivers based on monolithic microwave integrated circuit (MMIC) technology, the all-electric receiver based on envelope detection does not require the generation of a local oscillator signal related to the mmWave carrier frequency. The core task of the envelope detector is to determine the amplitude variations of the intensity-modulated signal. Meanwhile, this approach avoids complex frequency synthesis or mixing operations, making it suitable for low-complexity scenarios. In contrast, the all-optical receiver employs an ultra-wideband mmWave electro–optic modulator to directly modulate the received mmWave signal onto the optical carrier. Subsequently, an optical band-pass filter filters out one of the sidebands as the optical baseband signal [4,5].

**Figure 1:**
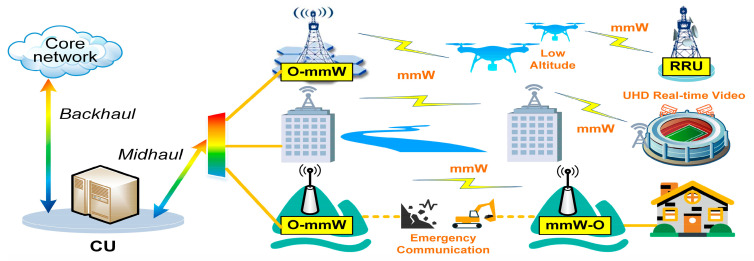
Scenarios for mmWave wireless communication under IMT-2030 framework.

Under the IMT-2030 framework, particularly within the context of the newly defined usage scenarios, such as immersive communication, massive communication, and ubiquitous connectivity, the demand for emerging applications like uncompressed 4K/8K ultrahigh-definition (UHD) video has surged, leading to an exponential increase in data traffic [6]. Optical-to-millimeter-wave (O-mmW) and millimeter-wave-to-optical (mmW-O) conversions enable the seamless integration of millimeter-wave wireless communication with existing optical fiber networks. As shown in Figure 1, this approach allows for high-performance and cost-effective transmission in scenarios such as low-altitude unmanned aerial vehicle (UAV) wireless relays, locations where laying optical fibers is impractical (e.g., across rivers), and emergency communications during fiber-optic disruptions caused by disasters.

To achieve the aforementioned objectives, one trend in the evolution of 5G and future 6G communications is the use of high-order modulation formats and offline digital signal processing [7,8]. However, this scheme increases receiver complexity and latency, thereby hindering real-time wireless transmission applications. To resolve this issue, at the transmitting side, employing photonics-assisted heterodyne beating techniques is an effective technical solution for generating wideband, high-quality mmWave and terahertz signals. On the receiving side, leveraging advancements in detectors based on Schottky barrier diodes (SBD) for mmWave and terahertz signal detection [9], one practical approach is to utilize the simple architecture of the all-electric receiver based on envelope detection. Another viable approach is to select a suitable high-speed electro–optic modulator, utilizing an all-optical receiver to improve transmission performance while balancing system complexity with the benefits of reduced insertion loss and spurious noise. The key devices in the all-electric receivers and all-optical receivers are envelope detectors and photodetectors, respectively. They are commercially available and have been continuously upgraded, enabling the demonstration of mmWave wireless communication systems with increasingly higher bandwidth and data rates. For instance, a balanced Schottky diode envelope detector, capable of demodulating amplitude shift keying (ASK) signals, is used to demonstrate mmWave transmission at data rates up to 14 Gbit/s, with a tunable carrier frequency between 78 GHz and 92 GHz [10]. On the other hand, although the all-optical receiver is more complex in structure and higher in cost compared to the all-electric receiver, it can achieve significantly higher data rates. As the key component of the all-optical receiver, avalanche photodiodes (APDs) are increasingly being used in wireless communication systems at the mmWave and Terahertz bands, particularly in scenarios where their ability to handle weak signal detection and high-speed data is crucial [11]. For example, a system using APD on the receiving side achieves error-free transmission at data rates between 30 Gbit/s and 50 Gbit/s over a distance of 10 m at a carrier frequency of 300 GHz [12]. However, there has been no simultaneous demonstration and comparison of the transmission performance of an all-electric receiver based on envelope detection and an all-optical receiver based on APD, which is crucial for providing valuable guidance on balancing cost and transmission performance, as well as on practical applications like transmitting uncompressed HD or UHD 4K/8K video.

This paper experimentally demonstrates the mmWave transmission system with an all-electric receiver based on envelope detection and an all-optical receiver based on APD. Firstly, we investigate the transmission performance of the two receiving schemes at a 100 GHz carrier frequency and a 15 m wireless link. The all-optical receiver scheme achieves error-free transmission of an 11.318 Gbit/s OOK signal, while the all-electric receiver based on envelope detection can only achieve error-free transmission up to 3.125 Gbit/s for the OOK signals. Furthermore, in the exploration of long-distance transmission using the all-optical receiver scheme, we achieve 200 m of error-free transmission at 10 Gbit/s. Additionally, to verify the feasibility of this scheme in practical applications, we accomplished real-time transmission of uncompressed HD video over a distance of 200 m and 2 km. The demonstrated systems reveal that the all-optical receiver is a more promising millimeter-wave-receiving approach for supporting future 6G scenarios requiring ultra-wideband, high-capacity, and wide-coverage high-speed wireless communications.

## 2. Link Budget Analysis

To provide guidance for subsequent wireless transmission experiments, it is necessary to conduct a theoretical analysis of transmission loss and perform the link budget. As is well known, the free-space path loss in wireless transmission is proportional to the square of the frequency. Therefore, the free-space loss in the transmission at the mmWave band is significantly higher than that at the lower frequencies currently used by operators. Thus, conducting theoretical analysis and calculations of transmission loss and power budget before performing specific experiments is of great importance in guiding the selection of experimental parameters.

According to the Friis transmission equation [13,14], the relationship between the transmitted power $P_{T}$, received power $P_{R}$, transmitter antenna gain $G_{R}$, receiver antenna gain $G_{T}$, and the path loss is given as follows:

(1) $$P_{R}=P_{T}+G_{T}+G_{R}-\bar{L_{P}}\left(d\right)-L_{f}-L_{a}\times d,$$

(2) $$\bar{L_{P}}\left(d\right)=L_{FS}\left(d_{0}\right)+10n\log\left(\frac{d}{d_{0}}\right),$$

where *d* is the distance of wireless transmission, $\bar{L_{P}}\left(d\right)$ is the average path loss at a specific distance, $L_{FS}\left(d_{0}\right)$ is the free-space path loss at the reference distance, expressed as $L_{FS}\left(d_{0}\right)=20\log\left(\frac{4\pi d_{0}}{\lambda }\right)$, where λ is the wavelength, and the typical reference distance is $d_{0}=1\;m$; n is the path loss exponent, which represents the rate at which the path loss increases with the distance between the transmitter and receiver, $L_{f}$ is the line loss, and $L_{a}$ is the atmospheric attenuation factor, which can be described by the specific attenuation calculated based on atmospheric conditions.

In the aforementioned theory, the ITU-R P.676-12 recommendation provides the method for calculating atmospheric attenuation; this method cumulatively accounts for the individual spectral lines of oxygen and water vapor under any pressure, temperature, and humidity conditions. It also considers factors such as the non-resonant Debye spectrum of oxygen below 10 GHz, nitrogen attenuation above 100 GHz mainly caused by pressure, and the moist continuum band, which results from excessive water vapor absorption observed in computational experiments [15]. Based on the factors considered in this recommendation, we have adjusted the parameters of the ITU atmospheric loss model using meteorological data from the past 30 years, including annual average relative humidity, atmospheric pressure, and temperature in Nanjing, Jiangsu Province, China, where the experiments are conducted. This makes the atmospheric loss model more closely aligned with the experimental environment of this work. The parameters of these two cases are shown in Table 1. Case 1 refers to the meteorological condition parameters provided in the standard example from the ITU-R P.676-12 document, while Case 2 represents the typical values corresponding to the historical annual meteorological data for Nanjing, as described earlier.

**Figure 2:**
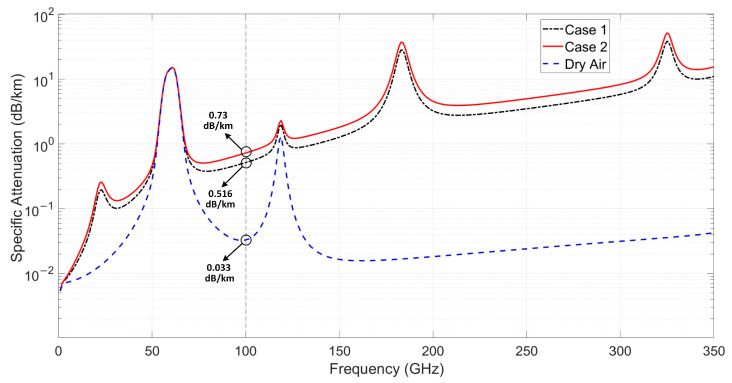
Specific attenuation as a function of frequency under case 1, case 2, and dry air condition.

Figure 2 shows the specific attenuation curve under case 1 and case 2, as well as the specific attenuation curve under dry air conditions, where the absolute humidity is set to zero, for reference. Under dry air conditions and Case 1, the specific attenuation at 100 GHz is 0.033 dB/km and 0.516 dB/km, respectively. Under the typical meteorological conditions in Nanjing (Case 2), the specific attenuation at 100 GHz is 0.73 dB/km, while it will increase to over 50 dB/km at frequency bands above 300 GHz under certain weather conditions. Therefore, for short-range communication in the W-band, the atmospheric attenuation can be almost negligible, but, as the frequency increases and the distance extends, the atmospheric attenuation will significantly affect the transmission performance [16].

According to Equation (2), the free-space path loss for transmission over 200 m and 2 km at a carrier frequency of 100 GHz is 118.46 dB and 138.46 dB, respectively. In designing the experimental setup, incorporating the precise atmospheric attenuation and free-space path loss into Equation (1) enables the rational selection of experimental parameters such as transmit power, transmit antenna gain, and receive antenna gain, considering achievable receiver sensitivity. This is particularly crucial for effectively conducting long-distance transmission experiments in the millimeter-wave and terahertz frequency bands. The specific details of the power budget will be provided later in this paper.

## 3. Scenario Validation

### 3.1. Experimental Setup

Based on the aforementioned analysis, we construct corresponding experimental platforms for scenario validation. Table 2 summarizes the general experimental setup and relevant parameters of the experiments in this work.

The three experiments for scenario validation, as shown in the table, all employ a transmitter architecture based on the photonics-assisted heterodyne beating technique, generating the mmWave with a frequency of 100 GHz. Indoors, we test the transmission performance at a distance of 15 m using the all-optical receiver and the all-electric receiver based on envelope detection at the receiving end. During the measurement process, bit error rate testers (BERTs) are used to generate test signals and evaluate the quality of the transmitted signals. The phase noise is measured by analyzing the frequency stability of the signals using a high-precision signal and spectrum analyzer. Eye diagrams for all experiments are measured using an oscilloscope and an eye diagram analyzer, providing visual assessments of transmission quality along with quantitative jitter analysis, enabling further evaluation and comparison of transmission performance under different schemes and experimental conditions. Outdoors, to further explore the advantages of the all-optical receiver in long-distance transmission and practical application, we test the transmission performance at distances of 200 m and 2 km and demonstrate real-time uncompressed HD video transmission.

**Figure 3:**
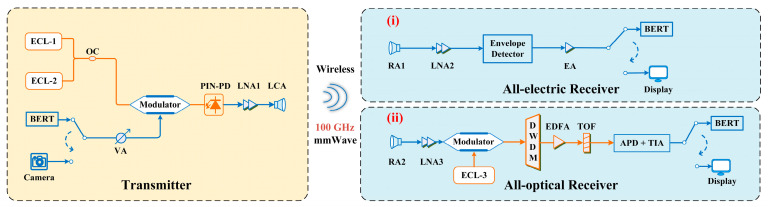
The architecture of the transmission system, including approaches of the all-electric receiver based on envelope detection and all-optical receiver. (**i**) All-electric receiver. (**ii**) All-optical receiver.

**Figure 4:**
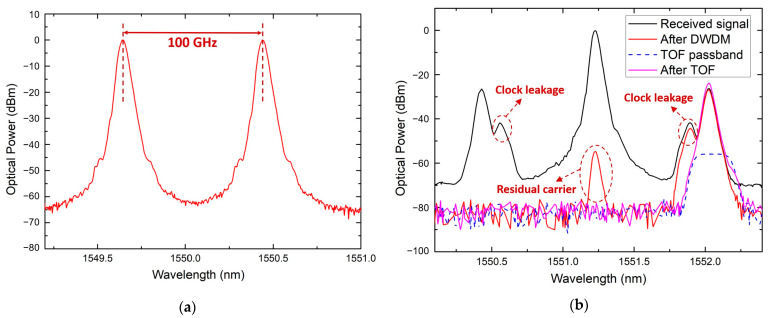
The measured optical spectra: (**a**) after the intensity modulator at the transmitting side, (**b**) of the received signal after the phase modulator, DWDM, TOF in the all-optical receiver, and the passband of TOF.

Figure 3 shows the architecture of the transmission system in this work. The transmitter employs the photonics-assisted heterodyne beating technique to modulate the OOK signals generated by the BERT or the real-time data stream of a camera onto the optical carrier, thereby generating and transmitting millimeter-wave signals. The receiver is implemented via two approaches: (i) an all-electric receiver based on envelope detection and (ii) an all-optical receiver based on an avalanche photodiode. The measured optical spectrum after the modulator at the transmitting side is shown in Figure 4a; the measured optical spectra of the received signal after the modulator, dense wavelength division multiplexing (DWDM), the tunable optical filter (TOF) in the all-optical receiver, and the passband of TOF are shown in Figure 4b. The parameters of key components used in the experiment are provided in Table 3.

### 3.2. Calculation of Power Budget in the Wireless Link

Table 4 presents the actual weather conditions during the 200 m and 2 km outdoor transmissions. Using the parameters recorded in the table, the ITU P.676-12 model can be applied to further refine the atmospheric loss values, enabling a more accurate power budget. Table 5 lists the power budget in the wireless link of outdoor scenarios based on the aforementioned actual meteorological conditions, the received power reaches −37.835 dBm and −45.352 dBm at transmission distances of 200 m and 2 km, respectively.

### 3.3. Indoor 15 m Transmission Using All-Optical and All-Electric Receiver

**Figure 5:**
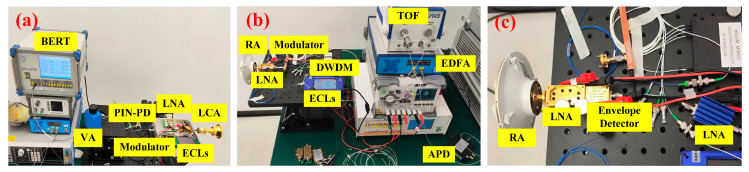
Photos of the indoor transmission system: (**a**) transmitter, (**b**) all-electric receiver, (**c**) all-optical receiver.

Figure 5 provides photos of the indoor transmission system; to ensure a fair and reasonable comparison, we use the same structure based on photonics-assisted heterodyne beating techniques at the transmitting side of the mmWave transmission experimental system. Additionally, the transmitting antennas, receiving antennas, and other components are consistently kept identical throughout the indoor experiments.

On the transmitting side, a BERT is employed to generate OOK signals with modulation frequencies corresponding to data rates ranging from 1.25 Gbit/s to a maximum of 11.318 Gbit/s. The modulation bandwidth is determined by the data rate and spectral efficiency of the OOK signals. For instance, a data rate of 11.318 Gbit/s corresponds to a modulation bandwidth of approximately 11.318 GHz. The BERT is configured to generate signals using a pseudorandom binary sequence of length 31 (PRBS31), with the signal amplitude adjustable between 200 mV and 500 mV. The transmission link is carefully calibrated before each experiment, and the BERT configuration parameters are double-checked to ensure consistency. Two external cavity lasers (ECL-1 and ECL-2), each with a linewidth of less than 100 kHz, are utilized to generate the optical carrier. The wavelengths of the two aforementioned lasers are set to 1549.190 nm and 1549.990 nm, respectively, corresponding to a wavelength interval of 0.8 nm, which means that the frequency of the mmWave signal generated by the heterodyne beating of the two laser beams is 100 GHz. The signal from the BERT is first attenuated using a variable attenuator (VA) with manually adjustable attenuation levels ranging from 0 to 9 dB before being sent to an optical intensity modulator. This modulated signal, combined with another optical signal generated by ECL-2 using an optical coupler (OC), is transmitted through a single optical fiber. Subsequently, these two signals undergo optical heterodyne beating in a PIN photodiode (PIN-PD), generating a 100 GHz mmWave signal, which is then amplified by a low-noise amplifier (LNA) and transmitted through a lens-corrected antenna.

On the receiving side, we employ experimental system structures based on two different approaches. For the all-electric receiver based on envelope detection, the mmWave signal received by the RA is amplified by an LNA and then input into a W-band envelope detector (AT-PD-75110N1, AT Microwave Limited, in Shanghai, China) for envelope detection. The envelope detector extracts the amplitude information of the modulated signal through nonlinear rectification and removes the carrier and high-frequency components using a low-pass filter. The output is the baseband component, which corresponds to the OOK signal, with a bandwidth identical to that set by the BERT at the transmitter. After being amplified by another LNA, the signal is fed into a BERT at the receiving side to test the BER performance. For the all-optical receiver, the mmWave signal is first received by a reflective antenna (RA), then amplified using a low-noise amplifier (LNA), and subsequently fed into an optical phase modulator. A laser from ECL-3, with a wavelength of 1550.800 nm, is introduced into the other port of the optical phase modulator. This step directly modulates the received mmWave signal onto the optical carrier, generating two optical sidebands. Afterward, a dense wavelength division multiplexing module is used to filter one of the optical sidebands, which acts as the optical baseband signal. This optical baseband signal is then amplified in an erbium-doped fiber amplifier (EDFA), filtered by a tunable optical filter, and sent into an APD for photoelectric conversion. The APD we selected has a bandwidth of 10 GHz, working at the wavelength range of 1250–1610 nm, and achieves a detection sensitivity of −15 dBm. The resulting signal is then amplified by a transimpedance amplifier (TIA) and input into a BERT to measure the transmission performance.

### 3.4. Outdoor 200 m and 2 km Uncompressed HD Video Transmission

**Figure 6:**
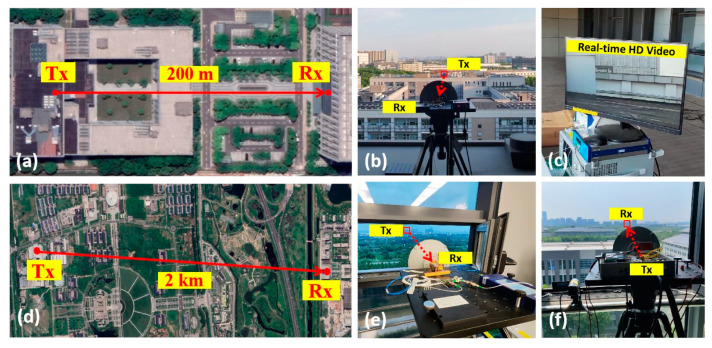
Experimental setup of the outdoor transmission: satellite map of the transmission link over a distance of (**a**) 200 m and (**d**) 2 km. Photos of the receiving side of (**b**) 200 m and (**e**) 2 km transmission. Photos of (**c**) real-time HD video displayed on the screen and (**f**) the transmitting side of 2 km transmission.

To explore the potential for long-distance transmission, we use the transmission system architecture based on an all-optical receiver to carry out the outdoor experiment. We replace the antennas of the aforementioned indoor demonstration system with larger aperture reflector antennas. A power amplifier (PA) is additionally added before the transmitting antenna for the 2 km transmission. This commercially mature PA operates at frequencies ranging from 75 to 110 GHz, with a typical gain of 16 dB. The rest of the experimental setup is kept unchanged. Then, we move the wireless link outdoors and extend the transmission distance from 15 m to 200 m and 2 km. The experimental setup for the outdoor transmission is shown in Figure 6.

The BER performance is first tested using BERTs. Additionally, simultaneously with long-distance transmission experiments, we also conduct practical application verification by replacing the signal source at the transmitter with a camera and substituting the BERT at the receiver with a display, thereby achieving live streaming of uncompressed HD video. A commercial camera is used in our experiment, and the real-time video captured by the camera is first input into a high-definition multimedia interface (HDMI) to serial digital interface (SDI) converter at the transmitting end. This converter is a high-quality, compact commercial device designed for converting HDMI video signals to professional SDI outputs. The SDI interface, supports a data rate of 1.485 Gbit/s, which is commonly used for uncompressed HD video transmission in broadcast-grade applications and corresponds to a modulation bandwidth of approximately 1.485 GHz in the wireless link when using OOK modulation. This aligns with the requirements for uncompressed HD video transmission. The SDI output interface transmits the video signal via a cable into the intensity modulator for modulation. The subsequent processes at the transmitting side, such as heterodyne beating in the PIN-PD, are identical to the procedures of indoor experiments. Similarly, on the receiving side, the baseband signal output from the APD is first input into an SDI to HDMI converter. The HDMI output interface of this converter is directly connected to a display, allowing the real-time video captured by the camera at the transmitting side to be displayed.

## 4. Experimental Results and Discussion

For both indoor and outdoor transmission experiments, we first measure the bit error rate (BER) performance under different data rates. In the indoor 15 m transmission experiment, by measuring the phase noise and eye diagram, we demonstrate that the all-optical receiver-based architecture offers superior performance for high-rate millimeter-wave wireless communication. Furthermore, in outdoor transmission experiments over distances of 200 m and 2 km, we explore the transmission performance of the proposed all-optical transceiver in long-distance scenarios and successfully demonstrate uncompressed HD video transmission.

### 4.1. Indoor Transmission Results

**Figure 7:**
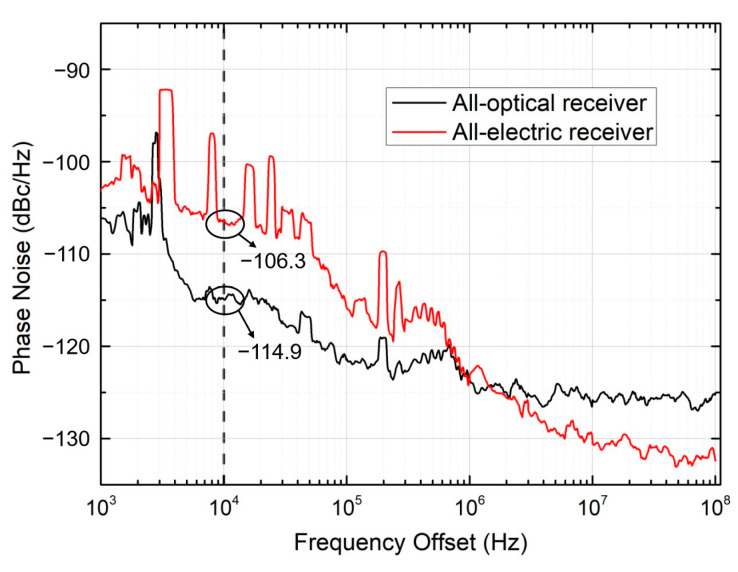
Phase noise as a function of frequency offset.

To investigate the underlying mechanisms behind the performance differences between the all-electric receiver based on envelope detection and the all-optical receiver based on APD, the phase noise of the two schemes is measured in our experiment. Figure 7 shows the phase noise as a function of frequency offset. This curve is obtained under the BtB configuration of a 1 GHz sine wave generated by an arbitrary waveform generator (AWG). Within the critical frequency offset range below 10^6^ Hz, the phase noise of the all-optical receiver is significantly lower than that of the all-electric receiver. Specifically, in the range of from 10 kHz to 100 kHz, the phase noise of the all-optical receiver is approximately 8 dB lower.

**Figure 8:**
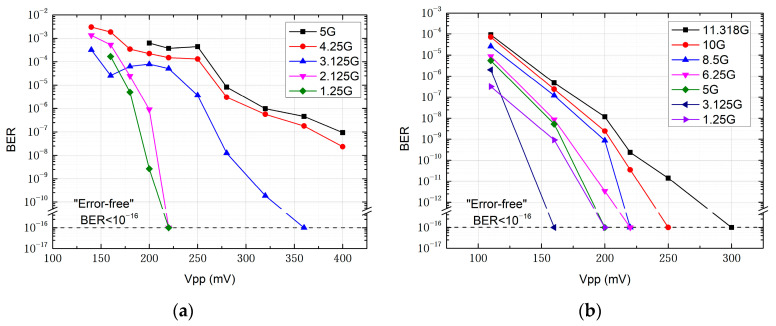
BER as a function of the signal amplitude into the intensity modulator for indoor transmission over a distance of 15 m based on (**a**) the all-electric receiver and (**b**) the all-optical receive.

The transmission performance of the two schemes shows significant differences under the influence of phase noise, which is caused by the characteristics of the devices and the system principles. Figure 8 shows the BER as a function of signal amplitude input to the transmitting side intensity modulator for 15 m indoor transmission using the all-electric and all-optical receivers, respectively. For the all-electric receiver, due to the bandwidth limitation of the envelope detector, the receiving side BERT fails to recover the data when the transmission rate exceeds 5 Gbit/s, resulting in a transmission link interruption. In contrast, the all-optical receiver is capable of supporting transmission rates up to the maximum of 11.318 Gbit/s generated by the BERT. Note that the system employing the all-electric receiver achieves error-free transmission of OOK signals up to a maximum of 3.125 Gbit/s, whereas the system using the all-optical receiver is capable of achieving error-free transmission at 11.318 Gbit/s with a signal amplitude of 300 mV. It is worth noting that the aforementioned error-free transmissions refer to long-term stable transmission with no bit errors, as indicated by a bit error rate (BER) lower than the measurable limit of 1 × 10^−16^ on the BERT, and all experiments are conducted without clock recovery.

**Figure 9:**
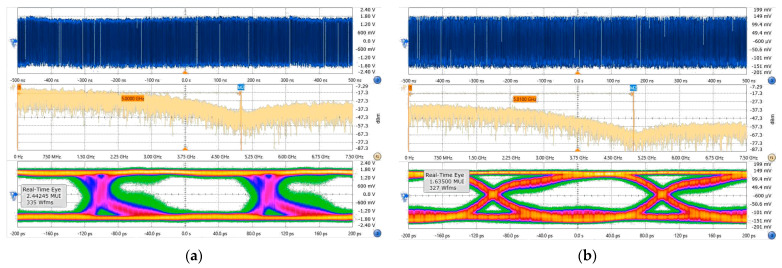
Signal waveform, spectrum, and demodulated eye diagram for 5 Gbit/s OOK signal of (**a**) all-electric receiver and (**b**) all-optical receiver.

To further evaluate the transmission performance, we also compare the quality of the eye diagrams for the two receiving schemes. Figure 9a,b show the waveform, spectrum, and demodulated eye diagrams for the 5 Gbit/s OOK signal, measured under the all-electric receiver scheme and the all-optical receiver scheme, respectively. In Figure 9a, we configured the oscilloscope to use the First Order PLL method for clock recovery in order to obtain a stable eye diagram; the bit error rate corresponding to the eye diagram is 6.8 × 10^−7^, making it challenging to achieve live streaming of uncompressed HD videos. Nevertheless, the all-optical receiver can achieve error-free transmission at a rate of 5 Gbit/s, and it can be seen in Figure 9b that the quality of the eye diagram without clock recovery is evidently better than that of the all-electric receiver. However, the rising and falling edges of multiple symbols are also no longer concentrated at a single position in the eye diagrams, appearing more dispersed. Quantitative analysis using an eye diagram analyzer reveals that this phenomenon is closely related to the larger jitter RMS value of 8.88 ps observed in the all-electric receiver, compared to the 7.07 ps jitter RMS in the all-optical receiver. The flicker noise and other factors inherent to the mechanism of the envelope detector are also key factors leading to signal degradation and transmission instability.

### 4.2. Outdoor Transmission Results

**Figure 10:**
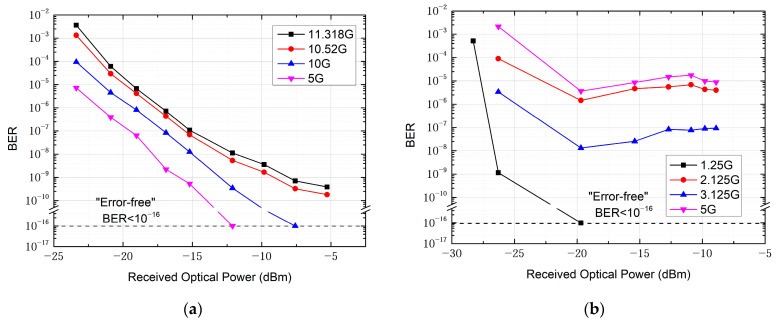
BER as a function of the received optical power for outdoor transmission over the distance of (**a**) 200 m and (**b**) 2 km.

Furthermore, we explore the capability of the communication system based on the all-optical receiver for long-distance transmission over distances of 200 m and 2 km, both of which successfully demonstrated real-time transmission of uncompressed HD video signals for practical scenarios. In the 200 m transmission, as indicated by the BER curve in Figure 10a, error-free transmission is achieved at data rates of 10 Gbit/s and below. For data rates of 10.52 Gbit/s and 11.318 Gbit/s, the bit error rate was on the order of 10^−9^ when the received optical power (ROP) of the APD on the receiving side approached −5 dBm. On the receiving side, we use an HD display to show the real-time video signal after transmission. The system maintains stable transmission for 24 h, with the bit error rate consistently below 10^−16^ and the video display free of noise. When the distance is further increased to 2 km, the system is still capable of stable transmission of uncompressed HD video. BER performance degrades as shown in Figure 10b; error-free transmission is achieved only at a rate of 1.25 Gbps. BER performance at 3.125 Gbps is superior to that at 2.125 Gbps, remaining on the order of 10^−8^. At 5 Gbps, BER performance deteriorates to the order of 10^−5^. These experimental results align with the theoretical estimate that the transmission rate required for uncompressed full HD video is around 3 Gbps, and they are consistent with the system characteristics observed in previous tests.

### 4.3. Stability and Power Consumption Evaluation

During indoor experiments, even in narrow corridors where multipath effects pose significant challenges, stable transmission is still achieved by carefully selecting appropriate antennas and transmission power. The scheme based on all-optical frequency up- and down-conversion demonstrates minimal jitter, with performance that is robust enough to support emerging 6G indoor applications, eliminating the need for clock recovery. For indoor real-time uncompressed video transmission, we continuously tested for 24 h, and, for outdoor transmission over 200 m and 2 km, we tested for 12 h, during which time the system maintained stable transmission. The experimental results from this work validate that the proposed transmission scheme based on all-optical up- and down-conversion has the potential to support future applications such as the real-time uncompressed transmission of UHD video.

Evaluating the power consumption of the experimental setup in this work is of significant importance in guiding the deployment of the system in practical applications. Based on calculations and measurements, the power consumption of the all-electric receiver and all-optical receiver in indoor experiments is approximately 2.1 W and 5.8 W, respectively. When normalized to the maximum achievable data rates under indoor conditions, the energy efficiency is approximately 0.67 W/Gbps for the all-electric receiver and 0.51 W/Gbps for the all-optical receiver. This indicates that the all-optical receiver is approximately 24% more energy efficient than the all-electric receiver, demonstrating its superior energy efficiency despite its higher absolute power consumption. For outdoor experiments, when adding a PA at the transmitter for long-distance transmission, the total power consumption of the communications system based on all-optical frequency up- and down-conversion is around 23.5 W. These findings highlight the trade-off between power consumption and energy efficiency in selecting the appropriate receiver approach for specific application scenarios.

## 5. Conclusions

We have experimentally demonstrated and compared the performance of all-optical and all-electric receivers in mmWave communication systems at 100 GHz. The all-optical receiver, utilizing an avalanche photodiode detector, achieves error-free 200 m transmission at data rates up to 11.318 Gbps, while the all-electric receiver, based on envelope detection, is limited to 3.125 Gbps error-free transmission over a 15 m wireless link. In addition to superior bit error rate performance, the mmWave communication system based on an all-optical transceiver demonstrates greater stability, lower phase noise, and a wider bandwidth. Moreover, the aforementioned system enables stable real-time transmission of uncompressed full HD video over distances of both 200 m and 2 km, with the all-optical receiver proving to be more effective for long-distance communication. The results suggest that the all-optical receiver is a more suitable choice for future millimeter-wave and terahertz communication systems that require high data rates and long-range transmission, as well as for applications such as uncompressed UHD video streaming in emerging 6G networks.

## Figures and Tables

**Table 1:** Different cases of meteorological conditions used in atmospheric attenuation model.

Case	Temperature	Atmospheric Pressure	Relative Humidity	Actual Vapor Pressure	Absolute Humidity	Specific Humidity
Case 1	15 °C	1013.25 hPa	-	-	7.5 g/m^3^	-
Case 2	16 °C	1017 hPa	74%	13.45 hPa	10.08 g/m^3^	13.85 g/kg

**Table 2:** Summary of the indoor and outdoor experimental setups.

Scenario	Carrier Frequency (GHz)	Receiver Scheme	Distance (m)	Antenna	PA ^3^ Used	BERT Test	Video Transmission
Indoor	100	All-optical/All-electric	15	LCA ^1^	×	√	Uncompressed HD
Outdoor	100	All-optical	200	RA ^2^	×	√	Uncompressed HD
Outdoor	100	All-optical	2000	RA	√	√	Uncompressed HD

^1^ LCA: Lens corrected antenna. ^2^ RA: Reflector antenna. ^3^ PA: Power amplifier.

**Table 3:** Parameters of key components in the experimental system.

Name	Parameter
LNA1	Frequency: 75–110 GHz Gain (Typical): 38 dB Noise Figure (Typical): 4 dB
LNA2, LNA3	Frequency: 75–110 GHz Gain (Typical): 32 dB Noise Figure (Typical): 4 dB
EA	Frequency: 10 MHz–50 GHz Gain (Typical): 35 dB Gain Flatness: ±2 dB
PIN-PD	Typical wavelength: 1550 nm Responsivity: 0.6 A/W 3 dB cut-off frequency: 100 GHz
APD + TIA	Bandwidth: 10 GHz Operating wavelength: 1250~1610 nm Responsivity: >4 A/W Sensitivity: −24.5 dBm Output voltage: 400 mV
Envelope Detector	Typical operating frequency: 75~110 GHz Sensitivity: 3000 V/W

**Table 4:** Actual weather conditions of outdoor transmissions.

Date	Distance (m)	Temperature (°C)	Atmospheric Pressure	Relative Humidity	Absolute Humidity	Actual Vapor Pressure	Specific Attenuation
29 May	200	27	1015 hPa	63%	13.88 g/m^3^	16.20 hPa	1.176 dB/km
2 September	2000	30	1010 hPa	78%	20.63 g/m^3^	23.64 hPa	1.901 dB/km

**Table 5:** Power budget of outdoor transmissions.

Parameter	Value	
Operation frequency	100 GHz	100 GHz
Distance	200 m	2 km
$P_{T}$	3.66 dBm	20.41 dBm
$G_{T}$	39 dBi	39 dBi
$G_{R}$	39 dBi	39 dBi
$\bar{L_{P}}\left(d\right)$	118.46 dB	138.46 dB
$L_{f}$	0.8 dB	1.5 dB
$L_{a}$	1.176 dB/km	1.901 dB/km
$P_{R}$	−37.835 dBm	−45.352 dBm

## Data Availability

The data presented in this study are available on request from the corresponding author. The data are not publicly available due to privacy restrictions.
